# A Moving Target: Firearm Deaths, Mental Health, and the Role of Physicians

**DOI:** 10.1007/s11920-024-01569-2

**Published:** 2024-12-11

**Authors:** Layla Soliman, Omari Baines-Waiz, John S. Rozel, Kelly Blankenship, James Rachal

**Affiliations:** 1 Atrium Health/Wake Forest School of Medicine, Wake Forest University-Charlotte Region, Charlotte, NC USA; 2 Atrium Health Carolinas Medical Center Sandra and Leon Levine Psychiatry Residency, Charlotte, NC USA; 3 UPMC Western Psychiatric Hospital, University of Pittsburgh, Pittsburgh, Pennsylvania USA; 4 Wright State University Boonshoft School of Medicine, Dayton, OH USA

**Keywords:** Suicide, Homicide, Firearm deaths, Mental illness

## Abstract

**Purpose of Review:**

This review aims to provide an updated overview of trends in firearm- related deaths, the mental health impact on communities, and clinical and legislative interventions. We examine existing interventions and highlight lesser-known yet impactful strategies, such as incorporating appropriate training in medical education on firearm safety. Additionally, we explore the broader impacts of firearm violence on community mental health and address the disputed topic of whether mental illness is a driving factor behind mass shootings.

**Recent Findings:**

The rate of firearm suicides has continued to grow in the United States, even as firearm homicides have slightly declined. While the media often attributes mass shootings to the perpetrator having a mental illness, research indicates such symptoms only account for a small subset of shooters. Recent studies highlight the benefits of incorporating firearm safety into professional medical education, which can reduce barriers for healthcare providers when discussing safe storage practices with patients.

**Summary:**

While suicide risk is frequently assessed among individuals with mental illnesses due to their heightened risk, other factors should be considered by all clinicians, not just those in mental health. Similarly, certain mental health symptoms may play a role in a small fraction of gun violence, but other risk factors account for most violence risk. Despite the implementation of firearm restriction laws and increased access to mental health resources, gaps remain that must be addressed to reduce not only the rate of suicide by firearm, but also the mental burden the aftermath has on the community.

## Introduction

Firearm death has become a controversial topic in the United States, with media coverage often spotlighting mass shootings and homicides. While current prevention strategies focus primarily on reducing firearm access and expanding mental health resources, communities are asking for other solutions as firearm suicides grow. These issues have grown in recent years, with the COVID-19 pandemic and resulting impact on mental health coinciding with record numbers of firearm sales [1]. Although those with serious mental illnesses face a higher risk of suicide, suicide screening and clinical judgement should also target other high-risk populations, including heavy alcohol users and those with firearm access. The healthcare community must also prioritize providing the necessary care to those impacted by mass shootings and other gun related violence. Given the high mortality rate associated with firearms, all physicians, not just psychiatrists, should be adept in discussing firearm safety with their patients. In this article, we explore recent findings on firearm suicides and homicides, the relationship with mental illness, policies that can reduce these events, and the role of medical education in promoting firearm safety.

### Firearm Suicides

In 2021, data collected from the Centers for Disease Control and Prevention on violent deaths identified suicide as the eleventh leading cause of death in the United States (US), and the second leading cause of death among individuals aged 10 to 34 years. Firearms were involved in 54.3% of suicides [2]. Over the years, data has consistently reported firearms as the most common method of suicide, with the highest rates seen in the elderly, white males, youth, and most recently American Indians/Alaska Natives [2–4]. Alarmingly, firearm suicides have increased by 24% over the past twenty years [5]. In response to this trend, various stakeholders have attempted to implement interventions, including increasing mental health resources and limiting firearm access.

Studies indicate that individuals with a mental illness are up to eight times more likely to die by suicide than the general population [6]. Data on specific mental health diagnoses and their associated suicide risk, specifically by method, is limited. Nonetheless, some studies have linked an increased risk of firearm suicide to post-traumatic stress disorder (PTSD) and schizophrenia [7]. However, it is important to recognize that other populations without a mental health diagnosis are also at risk for suicide, particularly firearm-related suicide. Therefore, improving mental health access for those with a mental health diagnosis cannot be the sole strategy for reducing suicide rates. Zuriaga et al.’s meta-analyses, reported that individuals without a mental illness diagnosis were on average 55% more likely to use a firearm to end their life than those with a mental health diagnosis [7]. Additional research has shown that individuals who have not received mental health treatment are more likely to use a firearm for suicide than other methods, such as poisoning or hanging [8]. For people with severe and persistent mental illness, this may be attributable to limited access to firearms due to financial and legal constraints. Among the 84.4% of decedents for whom mental health information was available for the National Violent Death Reporting System (NVDRS), nearly half had a diagnosed mental illness at the time of death. However, this data is not further categorized by method of suicide, but rather grouped by general violent methods, which include firearms, hanging, strangulation, and suffocation. Nearly 70% of those who died by violent suicide methods had never been treated for a mental health or substance use disorder. It remains unclear whether the individuals in this study were stable with their mental health, as decompensation has been known to increase the risk of firearm suicide [5]. These findings suggest that efforts to mitigate suicide by firearms are needed beyond those with mental health disorders.

While access to a firearm is the most well-known risk factor for firearm-related suicide, other risk factors beyond mental illness diagnoses include a personal crisis within two weeks prior to death, physical health limitations, and a history of suicidal ideation [2]. Substance use, particularly alcohol, around the time of suicide is another common risk factor [5]. Alcohol use impairs one’s judgement and increases impulsivity, which can lead to poor decision making [7]. In 2021, data from the NVDRS showed that among 45.5% of decedents with recorded blood alcohol concentration (BAC), 65% had a BAC greater than 0.08 g/dL. Lange and colleagues used data from the NVDRS spanning from 2003 to 2020 to examine the relationship between consumption and the use of firearms for suicide. They found that 54% of males and 30% of females who used a firearm to end their life had a positive BAC (greater than or equal to 0.01 g/dL). An analysis of the data revealed an inverted U-shaped curve, indicating that as BAC increases, the probability of using a firearm for suicide also increases, up to a certain point. However, this probability decreased once BAC levels exceeded 0.40 g/dL for males and 0.30 g/dL for females [9]. Another study attempted to relate previous alcohol-related charges at the time of legally purchasing a firearm with the likelihood of firearm suicide among males in California. Their findings show that the highest probability of firearm-related suicide occurred two years after the individual’s first firearm purchase. The probability decreased as more time passed from the purchase date but remained elevated for men who had two or more alcohol-related arrests or guilty verdicts within one year prior to legally purchasing their first handgun [10]. Although Lange and colleagues’ data suggests that reducing alcohol consumption could decrease the probability of firearm-related suicides, their study did not include information on whether participants had a diagnosis of alcohol use disorder, which could be a better indicator of suicide risk. Despite this, clinicians should be vigilant in counseling patients who disclose heavy alcohol use and access to firearms.

Given the rising number of firearm suicides in the US and the high lethality of this method, there have been increased calls for policy changes and risk reduction strategies. Because most suicide attempts are impulsive [5], leaving little time for intervention, restricting access to firearms has emerged as a potential method for reducing risk. However, efforts to limit firearm access must consider the legal implications of the Second Amendment. Under the Gun Control Act of 1968, individuals convicted of a felony or involuntarily committed to a mental health facility are prohibited from purchasing firearms [11]. Aside from these small groups, there is a larger population at risk of suicide. To address this, many states have implemented extreme risk protection orders (ERPOs), which allow law enforcement officers and family members to petition the court to temporarily remove firearms from an individual who is deemed to be a danger to themselves or others. Connecticut and Indiana were the first states to enact ERPOs, which have been associated with a reduction in firearm suicides, with an estimation of one life saved for every 10 to 20 firearm removals [6]. The court determines the duration of the firearm removal, with the maximum time before re-petitioning being one year. While the ERPO is active, the person cannot possess or purchase firearms. Although law enforcement officers are permitted to petition the court in all states with ERPOs, some states do not allow clinicians to directly petition the court, despite their critical role in assessing safety risks and counseling patients [11]. Therefore, expanding the ability of mental health providers to petition for ERPOs might enable earlier intervention.

As previously noted, while individuals with a mental illness have an increased risk of suicide, those without a documented mental illness are just as likely, if not more likely, to end their lives with a firearm [7]. Therefore, it is imperative that physicians across specialties assess their patients’ risk for suicide to help reduce the rate of firearm-related suicides. Unfortunately, current suicide risk assessment tools do not adequately capture individuals at an acutely elevated risk but rather identify those with many risk factors who may not attempt suicide. For example, the Columbia-Suicide Severity Rating Scale (C-SSRS) was used in an emergency department to assess suicide risk but was found to have poor sensitivity, as many patients who screened negative within the previous 30 days subsequently died by suicide. Physicians should use screening data alongside their clinical judgement when considering the likelihood of suicide completion without an intervention. [11]

### Firearm Homicides

Although most firearm deaths are suicides, homicides tend to grab headlines and burn into the public consciousness and drive more of the political discourse around guns. In 2021, there were record high firearm homicide deaths, though the per capita rate remained below the peak seen in 1974. [12]

While the rate of firearm homicide deaths dropped in 2022 and again in 2023, the numbers still exceeded pre-pandemic levels [13] There are common themes in media coverage of homicides. Active shooter incidents are rare and account for less than 1% of gun homicides in 2021. Still, they dominate the news cycle when they occur, sparking debate and calls to action.

Firearm access and mental health tend to be themes in the public discourse following these tragedies, though media framing of an incident can be influenced by many factors, including demographic characteristics of the perpetrator. [14]

Calls to action usually include two themes, decreasing gun access and increasing mental health access. Often, these are discussed as a binary choice, as opposed to synergistic efforts. While interpersonal conflict and criminal activity tend to drive most gun deaths, mental illness tends to come up more in high-profile incidents. This contributes to a false perception that people with mental illness are at a substantial risk of violence, when in fact they are more likely to be victims than perpetrators.

However, what is counted as a mental illness, and the extent to which an individual’s actions are attributable to mental illness, makes the picture more complex when it comes to active shooter incidents. For example, while most researchers agree that people with symptoms of serious mental illness are overrepresented in the small pool of mass public shooters, multiple researchers have concluded that most perpetrators do not have such symptoms or diagnoses, as described by Lankford and Cowan in 2020 [15]. The authors of that review point out that the criteria used in prior reviews may lead to an underestimate of the role of mental illness in active shooter incidents. The authors cite relying only on formal diagnoses, counting only certain diagnoses, concern about stigma, inaccurate stereotypes about how mental illness presents, and varying definitions of "mass shooting" as potential sources of inaccuracy. Still, psychotic symptoms, which tend to be the type most frequently portrayed in movies, etc., are present in a small subset of mass shooters [16]. Having a mental illness doesn’t preclude unrelated motivators for violence such as revenge for a firing, for example. Peterson, et al. conducted an open-source investigation into the role of psychotic symptoms in 168 mass shootings carried out by 172 perpetrators. They concluded that psychotic symptoms “may have played a minor role in 11% of cases, a moderate role in 9% of cases, and a major role in 11% of cases.” The authors concluded that increased access to mental health services “may help prevent mass shootings in a minority of cases” of what is already a very low base rate event. [17]

Research continues to indicate that crises, suicidality, and recent humiliating events are common risk factors for completed attacks. [18–20].

Direct threats or leakage (statements of intent to attack made to a third party) often precede attacks and may be the “chief complaint” for a referral to a psychiatric emergency service or crisis center. Bystander inaction has been found to increase the risk of a completed attack by 15-fold [20]. The nexus of acute crises or stressors, suicidality, and low rates of overt psychiatric illness implies a potential opportunity for crisis services to identify and prevent some incidents of targeted violence [21]. The National Institute of Justice published data from 50 years of public mass shootings in 2022, and found that almost 70% of public mass shooters were suicidal before or during the event. The authors noted a higher prevalence of suicidality among younger shooters, with “K-12 students who engaged in mass shootings found to be suicidal in 92% of instances and college/university students who engaged in mass shootings suicidal 100% of the time.” [22]

The ThreatAID (Assessing Imminent Dangerousness) tool from the California Bullet Points project identifies seven basic steps for evaluation of threats of targeted violence in emergency settings; their website provides detailed information and fillable PDF templates for customizing resources to individual clinical settings [23]. The National Council for Mental Wellbeing’s updated report on mass shootings highlights expanding access to Behavioral Threat Assessment and Management Teams and the importance of psychiatric evaluation and, if indicated, treatment of people at risk for serious violence while acknowledging that most acts of mass violence are not driven by psychiatric illness [24].

### Impact of Gun Violence on Communities

While most of the public discourse centers on the impact of mental health on gun violence, the more salient discussion is the effect of gun violence on the mental health of survivors, communities, and the national psyche.

Vasan, et al. found an increase in mental health related emergency room visits in the 14 days following exposure to neighborhood firearm violence. The authors noted that the number of visits increased with increased proximity to a shooting and advocated for universal trauma-informed care in emergency departments. [25]. Several studies have demonstrated that people in disadvantaged neighborhoods experience higher rates of gun violence, worsening existing disparities. [26, 27]). Evidence points to adverse effects on childhood development that can hinder school performance and further limit opportunities for kids in these neighborhoods. [26]. Ideally, support from healthy adults would buffer some of these effects, but gun violence is associated with poorer overall physical and mental community health. [27]. This makes it more challenging to provide a safe and supportive environment for children. Interventions to mitigate these inequities include trauma-informed care, improved access to mental health care, and violence interruption programs.

More broadly, preventing children from accessing firearms and limiting access for some adults have both shown utility in reducing injuries and deaths.

## Laws Restricting Firearms Access

ERPOs show growing evidence for reducing rates of overall and firearm suicide. They have been elevated from no evidence to limited evidence in the most recent update of the Rand Gun Policy in America meta-analysis [28]. While there is some debate about the broader public health impact of ERPO laws, ERPO petitions can be an invaluable tool for the clinical management of high risk patients who do not meet the threshold for involuntary commitment or other interventions. [29] Case series reports on ERPO applications for people at risk for mass shootings are also promising, albeit challenging to study with more robust statistical analysis [30].

The 2022 Bipartisan Safe Communities Act (BSCA) includes federal funding to support state-level implementation of ERPO laws [31]. Technical assistance for states is available from the National ERPO Resource Center under the auspices of the Bureau of Justice Assistance [32]. Other pertinent elements of the BSCA include expanded funding for mental health services and violence prevention programs, extending existing prohibitions on misdemeanant domestic violence offenders to include offenders who did not cohabitate with victims (i.e., the “boyfriend loophole”), added mental health and juvenile justice records for background checks of purchasers under the age of 21, and a number of measures to address firearm sales, transfers, and trafficking [33].

Child Access Prevention Laws.

In 2019, firearms became the leading cause of death in American children and adolescents, surpassing motor vehicle accidents and demonstrating a marked racial disparity with Black adolescents more than 14 times as likely to be killed by guns than White adolescents [33]. In a study of gun owners with children, 70% of the parents report that they have taken steps to assure their children cannot access the firearms; 33% of those children report that they can access a loaded firearm in less than 5 min.[34] Recent studies continue to confirm prior findings that child access prevention laws reduce child and adolescent suicides and homicides [35, 36]. Child access prevention laws have some of the most robust evidence amongst all policy-linked reductions in suicide, violent crime, and accidental injuries and deaths [37].

The Role of Academic Medical Centers.

For years progress in firearm prevention has been stymied by debate between gun rights and gun control advocates, lack of funding for research, and stigma [38]. Physicians have largely been taught to have a reactive role in treating firearm injuries, as opposed to the active, preventative role taken in other arenas such as cardiovascular health, seatbelt / car seat safety, etc. The reluctance to change is enhanced by lack of experience, expertise and clear guidance about firearm safety from national organization like AMA etc. [39] As part of training, physicians learn how to discuss death, gender identity, sexual orientation, suicide, and other sensitive topics with cultural competence. The same should be taught about firearm safety [38]. Thirty-five percent of American adults live in household where firearms are present. [40] Evidence has shown that safe firearm storage decreases the risk of firearm suicide, homicide and unintentional injury. [40]. Firearm homicide is largely concentrated within urban communities historically most affected by discriminatory housing practices and other elements of structural racism. [41]. Economic distress, lack of opportunity, and inadequate mental health services have contributed to elevated risk of rural and military veteran firearm suicide rates. These vulnerabilities are compounded by increased access to firearms, which allow for rapid access to a high-lethality method of suicide in a time of crisis. Firearm fatalities increased by 34.9% from 2010 to 2020, [42] accounting for 400,000 deaths and 1.2 million emergency room visits. Initial national healthcare costs following firearm injury are estimates of over $700 million, followed by another $100 million related to readmissions within 6 months.

Many medical professional associations have identified firearm injury and death as a public health issue, and there is good evidence to support clinician involvement in firearm injury prevention [42, 43]. Evidence has shown that when a clinician engages in firearm safety discussions with patients, they are effective in improving families’ safe storage behaviors [42, 43]. However, few clinicians have received education on even basic firearm epidemiology, risk factors and prevention. Practicing physicians, residents, and medical students surveyed reported low rates of knowledge and confidence related to firearm safety counseling. [42, 43] In a survey of firearm education training, respondents who had prior firearm education in medical training reported fewer barriers in addressing the topic in practice. This would suggest that firearm injury prevention training is effective in empowering physicians to discuss firearm safety with their patients 40].

As part of suicide prevention, trainees should be educated on the potential for temporary transfer of firearms for patients at acutely elevated risk. Credibility may be gained when the physician is able to discuss and understand that in states where gun transfers may be tightly regulated, trusted individuals can take possession of key components like firing pin or trigger mechanism [38]. This all points to a significant opportunity for medical schools and residency to provide more education in firearm safety and injury prevention. A paper by Hoops,et al. discusses guidelines that highlight the impact of counseling on safe storage. They also discuss the impact of effective policies and practices. [42] The evidence supports efforts to incorporate evidence-based firearm injury prevention into post graduate curriculum and work toward developing a universally accepted curriculum. [42, 44]. Despite being identified as a significant public health risk firearm prevention continues to be grossly underfunded, receiving only 12.5 million in FY2020 out of total grants of 30.8 billion. [45] This has led to a lack of comprehensive data sources. [41] The loss of life with firearms injury and death incurs a substantial economic burden and profound emotional effect on family, friends and community. Many of these events are preventable, but adequate research funding will be essential to developing evidence-based medical education curricula, clinical practices, and policies. Academic medical centers play an essential role in firearm prevention as they are uniquely positioned at the forefront of clinical research and education of the next generation of physicians. We must work across specialties to advocate for the needed allocation of funding and other resources required to improve medical education, clinical practice and policies.

## Conclusions

Firearm sales have increased substantially during and since the COVID 19 pandemic. Firearm access at times when an individual may be in crisis (even absent a diagnosable mental illness) increases the risk for suicide and homicide. While homicide draws more media attention and debates about policy, most firearm-related deaths are suicides. Although a mental health diagnosis is a risk factor for suicide, data reports that many individuals completing suicide by firearms have no previous mental health diagnosis. The largest risk factor for completed suicide by usage of firearms is immediate access to a firearm. As such, physicians and advanced practice providers across specialties should screen patients for suicidal ideation and consider a discussion about whether there is a firearm in the home, and recommendations for safer storage. In cases where a patient's risk for suicide (or less likely homicide) is acutely elevated, a discussion about a trusted support temporarily moving or disabling the firearm can be helpful.

Mass public shootings are often wrongly attributed to mental illness in the media and politics. While psychotic or other serious psychiatric symptoms may be over-represented among mass shooters, such symptoms are either not present or not a major factor in most of these tragedies. Centering policy discussions around mental illness is not effective in decreasing violence, and only serves to stigmatize an already vulnerable population.

Firearm related deaths of all kinds impact community mental health, especially in communities with limited resources. Policy changes and risk reduction strategies surrounding firearm access and improved mental health access are needed. Strategies for reducing morbidity and mortality related to firearms may include expanding the scope of extreme risk protection orders (ERPO) and Child Access Prevention Laws and improving funding and resource allocation to academic medical centers to create education curricula for clinicians surrounding firearm safety discussions with patients. Academic medical centers are uniquely positioned to shape firearm-related education for medical students, residents, and other trainees. This step can be taken in the short term, while also advocating to expand funding for research.

## Data Availability

No datasets were generated or analysed during the current study.

## References

[1] Lang BJ, Lang M. Pandemics protests, and firearms. Am J Health Econ. 2021;7(2):131–63.

[2] Nguyen B. L. (2024). Surveillance for Violent Deaths—National Violent Death Reporting System 48 States the District of Columbia and Puerto Rico 2021. MMWR. Surveillance Summaries 73 10.15585/mmwr.ss7305a1 PMC11262823 38980822

[3] Price JH, Khubchandani J. Firearm suicides in the elderly: a narrative review and call for action. J Community Health. 2021;46(5):1050–8. 33547617 10.1007/s10900-021-00964-7 PMC7864138

[4] Chaudhary S, Hoffmann JA, Pulcini CD, Zamani M, Hall M, Jeffries KN, Goyal MK. Youth suicide and preceding mental health diagnosis. JAMA Netw Open. 2024;7(7):e2423996–e2423996. 39078631 10.1001/jamanetworkopen.2024.23996 PMC11289695

[5] Pallin R., & Barnhorst A. (2021). Clinical strategies for reducing firearm suicide. *Injury Epidemiology* 8(1) 10.1186/s40621-021-00352-8 PMC8489372 34607607

[6] Swanson JW. Preventing suicide through better firearm safety policy in the United States. Psychiatr Serv. 2021;72(2):174–9. 32878544 10.1176/appi.ps.202000317

[7] Zuriaga A, Kaplan MS, Choi NG, Hodkinson A, Storman D, Brudasca NI, Brini S. Association of mental disorders with firearm suicides: a systematic review with meta-analyses of observational studies in the United States. J Affect Disord. 2021;291:384–99. 34098496 10.1016/j.jad.2021.05.005

[8] Bond AE, Bandel SL, Rodriguez TR, Anestis JC, Anestis MD. Mental health treatment seeking and history of suicidal thoughts among suicide decedents by mechanism 2003–2018. JAMA Netw Open. 2022;5(3):e222101. 35285919 10.1001/jamanetworkopen.2022.2101 PMC9907334

[9] Lange S, Jiang H, Kaplan MS, Kim KV, Rehm J. Association between acute alcohol use and firearm-involved suicide in the United States. JAMA Netw Open. 2023;6(3):e235248–e235248. 36988957 10.1001/jamanetworkopen.2023.5248 PMC10061235

[10] Schleimer JP, Wright MA, Shev AB, McCort CD, Asif-Sattar R, Sohl S, Kagawa RM. Alcohol and drug offenses and suicide risk among men who purchased a handgun in California: a cohort study. Prev Med. 2021;153:106821. 34599927 10.1016/j.ypmed.2021.106821

[11] Vitiello E, Roskam K, Swanson J. Balancing the roles of clinicians and police in separating firearms from people in a dangerous mental health crisis: legal rules, policy tools, and ethical considerations. J Law Med Ethics. 2023;51(1):93–103. 37226741 10.1017/jme.2023.44 PMC10209969

[12] https://www.pewresearch.org/short-reads/2023/04/26/what-the-data-says-about-gun-deaths-in-the-u-s/ (accessed 8/10/2024)

[13] https://www.cdc.gov/firearm-violence/php/data-trends/firearm-homicide-trends.html (Accessed 8/10/2024)

[14] Silva J. R. (2021). The news media's framing of mass shootings: Gun access mental illness violent entertainment and terrorism. Russ. J. Econ. & L. 332

[15] Lankford A, Cowan RG. Has the role of mental health problems in mass shootings been significantly underestimated? J Threat Assess Manag. 2020;7(3–4):135.

[16] Brucato G, Appelbaum PS, Hesson H, Shea EA, Dishy G, Lee K, Girgis RR. Psychotic symptoms in mass shootings v. mass murders not involving firearms: findings from the Columbia mass murder database. Psychol Med. 2022;52(15):3422–30. 10.1017/S0033291721000076 33595428

[17] Peterson JK, Densley JA, Knapp K, Higgins S, Jensen A. Psychosis and mass shootings: A systematic examination using publicly available data. Psychol Public Policy Law. 2022;28(2):280–91.

[18] West SJ, Thomson ND. Exploring personal crises observed in mass shooters as targets for detection and intervention using psychometric network analysis. Psychol Violence. 2023;13:415–24.

[19] Brucato G, Hesson H, Dishy G, Lee K, Pia T, Syed F, et al. An analysis of motivating factors in 1,725 worldwide cases of mass murder between 1900–2019. The Journal of Forensic Psychiatry & Psychology. 2023;0:1–14 10.1080/14789949.2023.2208570 PMC10435045 37600153

[20] Gibson KA, Craun SW, Ford AG, Solik K, Silver J. Possible attackers? A comparison between the behaviors and stressors of persons of concern and active shooters. J Threat Assess Manag. 2020;7:1–12.

[21] Rozel JS, Soliman L. 2024 Lessons of the Boom: A Playbook for Crisis Centers to prevent, survive, and respond to active assailants, targeted violence, and mass violence. Psychiatric Clinics [Internet]. 47. Available from: https://www.clinicalkey.com/#!/content/journal/1-s2.0-S0193953X24000273 10.1016/j.psc.2024.04.005 39122345

[22] Public Mass Shootings: Database Amasses Details of a Half Century of U.S. Mass Shootings with Firearms Generating Psychosocial Histories | National Institute of Justice

[23] Threat AID Tool [Internet]. BulletPoints Project. [cited 2024 Aug 5]. Available from: https://www.bulletpointsproject.org/threat-aid-protocol/

[24] Mass Violence in America: Causes, Impacts and Solutions (thenationalcouncil.org) (accessed on 8/22/2024)

[25] Vasan A, Mitchell HK, Fein JA, Buckler DG, Wiebe DJ, South EC. Association of neighborhood gun violence with mental health–related pediatric emergency department utilization. JAMA Pediatr. 2021;175(12):1244–51. 34542562 10.1001/jamapediatrics.2021.3512 PMC8453357

[26] Holloway K, Cahill G, Tieu T, Njoroge W. Reviewing the literature on the impact of gun violence on early childhood development. Curr Psychiatry Rep. 2023;25(7):273–81. 37233973 10.1007/s11920-023-01428-6 PMC10213564

[27] Semenza DC, Stansfield R, Silver IA, Savage B. Reciprocal neighborhood dynamics in gun violence exposure, community health, and concentrated disadvantage in one hundred US cities. J Urban Health. 2023;100(6):1128–39. 37843742 10.1007/s11524-023-00796-x PMC10728405

[28] Smart R, Morral AR, Murphy JP, Jose R, Charbonneau A, Smucker S. The Science of Gun Policy: A Critical Synthesis of Research Evidence on the Effects of Gun Policies in the United States, Fourth Edition [Internet]. 4th ed. Santa Monica CA: RAND Corporation; 2024 [cited 2024 Jul 17]. Available from: https://www.rand.org/pubs/research_reports/RRA243-9.html 10.7249/rhq-12-01-03 PMC11630101 39664971

[29] Swanson JW. Preventing Firearm Tragedies by the Numbers—Remembering Why It Matters. JAMA Netw Open. 2024;7:e2414842. 38865131 10.1001/jamanetworkopen.2024.14842

[30] Zeoli AM, Frattaroli S, Barnard L, Bowen A, Christy A, Easter M, et al. 2022 Extreme risk protection orders in response to threats of multiple victim/mass shooting in six U.S. states: A descriptive study. Preventive Medicine. 165:107304 10.1016/j.ypmed.2022.107304 36265579

[31] United States: National Archives and Records Administration: Office of the Federal Register. 2022 Public Law 117 - 159 - Bipartisan Safer Communities Act [Internet]. U.S. Government Publishing Office. [cited 2024 Aug 7]. Available from: https://www.govinfo.gov/app/details/PLAW-117publ159

[32] The National ERPO Resource Center [Internet]. The National ERPO Resource Center. [cited 2024 Aug 7]. Available from: https://erpo.org/

[33] Andrews AL, Killings X, Oddo ER, Gastineau KAB, Hink AB. Pediatric Firearm Injury Mortality Epidemiology. Pediatrics. 2022;149:e2021052739. 35224633 10.1542/peds.2021-052739

[34] Salhi C, Azrael D, Miller M. Parent and Adolescent Reports of Adolescent Access to Household Firearms in the United States. JAMA Netw Open. 2021;4:e210989. 33687444 10.1001/jamanetworkopen.2021.0989 PMC7944379

[35] Kivisto AJ, Kivisto KL, Gurnell E, Phalen P, Ray B. Adolescent Suicide, Household Firearm Ownership, and the Effects of Child Access Prevention Laws. J Am Acad Child Adolesc Psychiatr. 2021;60:1096–104. 10.1016/j.jaac.2020.08.442 32971189

[36] Mark Anderson D, Sabia JJ, Tekin E. Child access prevention laws and juvenile firearm-related homicides. J Urban Econ. 2021;126:103387. 34898733 10.1016/j.jue.2021.103387 PMC8664083

[37] Smart R, Morral AR, Murphy JP, Jose R, Charbonneau A, Smucker S. 2024 The Science of Gun Policy: A Critical Synthesis of Research Evidence on the Effects of Gun Policies in the United States [Internet]. 4th ed. Santa Monica CA: RAND Corporation. [cited 2024 Jul 17]. Available from: https://www.rand.org/pubs/research_reports/RRA243-9.html. 10.7249/rhq-12-01-03 PMC11630101 39664971

[38] Betz ME, Harkavy-Friedman J, Dreier FL, Pincus R, Ranney ML. Talking about “firearm injury” and “gun violence”: words matter. Am J Public Health. 2021;111(12):2105–10. 34878863 10.2105/AJPH.2021.306525 PMC8667825

[39] Barron A, Hargarten S, Webb T. Gun violence education in medical school: a call to action. Teach Learn Med. 2022;34(3):295–300. 33882766 10.1080/10401334.2021.1906254

[40] Pallin R, Teasdale S, Agnoli A, Spitzer S, Asif-Sattar R, Wintemute GJ, Barnhorst A. Talking about firearm injury prevention with patients: a survey of medical residents. BMC Med Educ. 2022;22:1–7. 34980095 10.1186/s12909-021-03024-9 PMC8725249

[41] Roche JS, Carter PM, Zeoli AM, Cunningham RM, Zimmerman MA. Challenges, successes, and the future of firearm injury prevention. Milbank Q. 2023;101(Suppl 1):579. 37096629 10.1111/1468-0009.12621 PMC10126989

[42] Hoops, K., Fahimi, J., Khoeur, L., Studenmund, C., Barber, C., Barnhorst, A., ... & Ranney, M. L. (2022). Consensus-driven priorities for firearm injury education among medical professionals. *Academic medicine*, *97*(1), 93–104 10.1097/ACM.0000000000004226 34232149

[43] Titus SJ, Huo L, Godwin J, Shah S, Cox T, Ogola GO, Ahmed KW. June). Primary care physician and resident perceptions of gun safety counseling. Baylor Univ Med Cent Proc. 2022;35:405–9. 10.1080/08998280.2021.2004532 PMC9196655 35754582

[44] Mueller KL, Blomkalns AL, Ranney ML. Taking aim at the injury prevention curriculum: educating residents on talking to patients about firearm injury. Acad Med. 2022;97(10):1433–7. 35442908 10.1097/ACM.0000000000004707

[45] CDC’s Firearm Violence and Injury Prevention Research and Activties (nih.gov) last accessed on 8/18/2024

